# Asthma guidelines: Global to local

**DOI:** 10.4103/1817-1737.56006

**Published:** 2009

**Authors:** J. Mark FitzGerald

**Affiliations:** *Professor of Medicine, Head of the UBC Respiratory Division and Director of the Centre for Lung Health, The Lung Centre, Vancouver General Hospital, 2775 Laurel Street, Vancouver BC V5Z1M9, Canada*

Asthma continues to have a major impact on public health globally. Although, fortunately, death from asthma is now infrequent, there is still an unacceptable level of morbidity and associated economic burden.[1] The first national evidence-based asthma treatment guidelines was published following a meeting in Canada in 1989. Since then the rigor of the methodology used to develop such guidelines has significantly increased. Guidelines serve a number of functions apart from defining an evidence-based standard of care. They prioritize the effectiveness of different interventions so that national dug formularies can identify the most cost-effective interventions. This is especially important as newer, more expensive, biologicals become available for the treatment of asthma. The process of defining levels of evidence also helps to identify, for clinician investigators, where the gaps are in the evidence required to make robust recommendations and thus highlight the need for specific new randomized controlled trials. The availability of evidence-based recommendations also creates an environment whereby traditional narrative reviews with no reported search strategy and unstructured recommendations can be more robustly critiqued. This process requires a significant infrastructure and is increasingly beyond the capacity of national organizations. The Global Initiative in Asthma (GINA) has evolved such an infrastructure and provides an evidence base which can be adapted by national or regional professional societies to create their own guidelines.[2]

The new Saudi Arabian asthma guidelines published concurrently with this editorial is an excellent example of global guidelines being adapted to local conditions.[3] The authors who have undertaken this task are to be congratulated. Although the fundamental therapeutic recommendations remain the same, when these are reframed by national ‘champions’ there is a greater likelihood that they will be successfully implemented locally. As the process for developing asthma guidelines has improved, the challenge of successfully implementing them has become more obvious. There have been notable examples where national guidelines have been very successful in dramatically changing the management of asthma in a very cost-effective manner.[4]

In the light of a recent Canadian study that showed significant over-diagnosis of asthma,[5] all patients should have the diagnosis of asthma confirmed. This should include the use of objective measures of airflow obstruction and, if necessary, assessment of airways hyperresponsiveness. The current guidelines continue to emphasize the importance of introducing early anti-inflammatory therapy, most commonly in the form of inhaled corticosteroids. In addition, all patients should receive structured education, including the provision of a written action plan. Inhaler device technique should be reviewed regularly, especially if patients are using multiple devices of different types. Patients uncontrolled on inhaled corticosteroids—and in whom issues around adherence, inhaler technique, and the treatment of comorbidities, especially rhinitis, has been addressed—should be considered as candidates for the addition of a long-acting beta agonist. Although concerns have been raised about the safety of long-acting beta agonists, there is a robust evidence base suggesting that they are effective and safe. The very large sample sizes—in the hundreds of thousands of patients—that have been calculated as being required to assess mortality risk would imply that such risk is low.

Montelukast maybe considered as an alternate initial therapy in patients who will not or cannot take inhaled corticosteroids. They are also an alternate add-on therapy, although with a less robust evidence base than that for long-acting beta agonists.

In summary, this new document provides physicians and allied health workers in Saudi Arabia with an excellent framework for the management of asthma. The challenge now is for these guidelines to be implemented successfully, so that the care gaps identified in the document can be closed and the quality of life for asthma patients can be improved.
